# A simple and effective “capping” approach to readily tune the fluorescence of near-infrared cyanines†
†Electronic supplementary information (ESI) available: Experimental procedures, characterization data, and additional spectra. See DOI: 10.1039/c5sc00348b



**DOI:** 10.1039/c5sc00348b

**Published:** 2015-05-05

**Authors:** Longwei He, Weiying Lin, Qiuyan Xu, Mingguang Ren, Haipeng Wei, Jian-Yong Wang

**Affiliations:** a Institute of Fluorescent Probes for Biological Imaging , School of Chemistry and Chemical Engineering , School of Biological Science and Technology , University of Jinan , Jinan , Shandong 250022 , P.R. China . Email: weiyinglin2013@163.com; b State Key Laboratory of Chemo/Biosensing and Chemometrics , College of Chemistry and Chemical Engineering , Hunan University , Changsha , Hunan 410082 , P.R. China

## Abstract

Heptamethine cyanines are favorable for fluorescence imaging applications in biological systems owing to their near-infrared (NIR) absorption and emission. However, it is very difficult to quench the fluorescence of NIR dyes by the classic photoinduced electron transfer mechanism due to their relatively high-lying occupied molecular orbital energy levels. Herein, we present a simple and effective “capping” approach to readily tune the fluorescence of NIR cyanines. The resulting new functional NIR **CyBX** (**X** = **O**, **N**, or **S**) dyes not only retain the intact tricarbocyanine scaffold, but also have a built-in switch to regulate the fluorescence by spiro-cyclization. When compared to traditional cyanines, novel **CyBX** dyes have a superior character in that their NIR optical properties can be readily tuned by the intrinsic spiro-cyclization mechanism. We expect that this “capping” strategy can be extended across not only the visual spectrum but also to structurally distinct fluorophores.

## Abstract 2

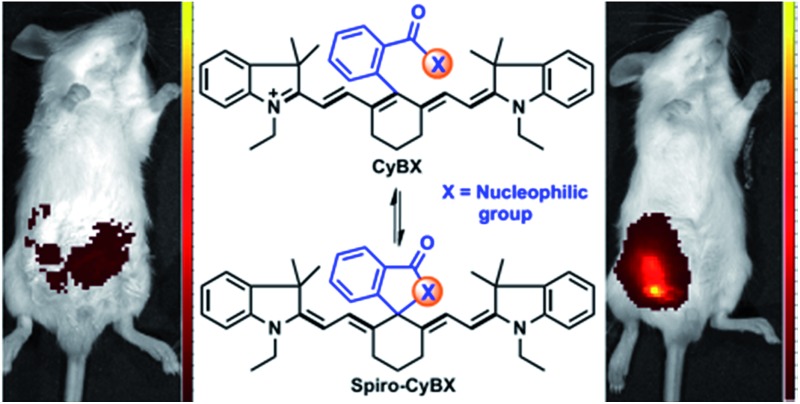
A simple and effective capping approach was introduced to readily tune the fluorescence of NIR cyanines.

## Introduction

In 1856, Williams first discovered cyanine dyes.^1^ The desirable features of cyanine dyes include narrow absorption bands and high molar absorption coefficients. Members of the cyanines such as monomethine and trimethine cyanines (Cy3) generally display absorption and emission only in the visible region. The extension of the chromophore backbone of cyanine dyes by one vinylene moiety (CH=CH) may lead to a bathochromic shift of ∼100 nm.^2^ For instance, the maximal emission wavelengths of pentamethine (Cy5) and heptamethine cyanines (Cy7) can extend well into the near-infrared (NIR) region (>650 nm). However, increasing the length of the polymethine chain may elicit unwanted effects including low fluorescence quantum yields, poor photostability, and aggregation.^3^

With great efforts of the researchers in the field, these limitations have been addressed at least to a certain extent. For example, the introduction of a rigid chlorocyclohexenyl ring in the methine chain was employed to improve the stability and the fluorescence quantum yields of cyanine dyes.^4^ Maury *et al.* demonstrated that a small, hard anion could polarize the polymethine chain and therefore enhance the stability of cyanines.^5^ Blanchard's group reported that conjugation of the cyanine fluorophore Cy5 with a triplet-state quencher could significantly improve the photostability of the dye.^6^ Pham and co-workers described that tricarbocyanine modified with four water-soluble sulfonate groups could suppress aggregation.^7^

**Scheme 1:**
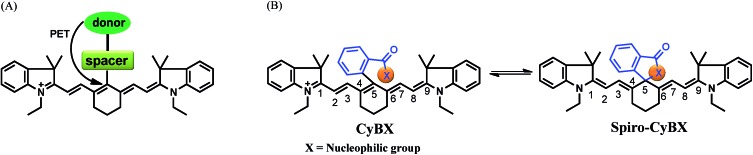
Design strategy for the new NIR functional **CyBX** (**X** = **O**, **N**, or **S**) dyes with an intrinsic spiro-cyclization based mechanism which modulates fluorescence On–Off. (A) The traditional PET mechanism for tuning fluorescence of heptamethine cyanines. (B) The spiro-cyclization based process of the novel functional NIR **CyBX** dyes described in this work.

NIR tricarbocyanine dyes are the most useful members of the cyanine dyes and have been applied as functional fluorescent dyes in diverse fields such as monitoring disease biomarkers, detecting biomolecules, monitoring variations of physiological environments, sensing enzyme activity, studying protein–DNA interactions, and evaluating drug efficacy, all due to the pronounced advantages of the NIR properties.^8^ Up to date, photoinduced electron transfer (PET) is the main fluorescence mechanism employed for modulating fluorescence of NIR tricarbocyanines (Scheme 1A).^8e,f,9^ However, it is very difficult to quench the fluorescence of NIR dyes by the PET mechanism due to their relatively high-lying occupied molecular orbital (HOMO) energy levels.^10^

This has, for a long time, constrained the full potential applications of this important class of NIR fluorescent dyes. Thus, it is crucial to introduce a new strategy to readily modulate the fluorescence of NIR cyanines.

Toward this end, in this contribution, we present a unique means to tune the fluorescence of NIR tricarbocyanines (Scheme 1B). Exemplified by heptamethine cyanine (Cy7), which is a widely used NIR dye, a carboxylic acid moiety (or its amide/thioic derivative) is strategically installed on the carbon 5-position (the central carbon) of the intact cyanine backbone to afford **CyBX**. We envisioned that **CyBX** could be transformed into **Spiro-CyBX** due to nucleophilic attack of heteroatom **X** on the electrophilic carbon 5. As **CyBX** essentially maintains the native scaffold of heptamethine cyanine, like heptamethine cyanine it should display absorption and emission in the NIR region. However, the typical “push–pull” characteristic of classic cyanines is disrupted in **Spiro-CyBX**, we thus anticipated that **Spiro-CyBX** may show almost no absorption and fluorescence in the NIR region. In other words, the carboxylic acid moiety (or its derivative) may function as a built-in mechanism to readily tune the fluorescence of heptamethine cyanine. Thereby, the transformation of non-fluorescent **Spiro-CyBX** into fluorescent **CyBX** could be exploited to design turn-on type NIR fluorescent probes for bioimaging applications in living systems.

Herein, we describe the rational design, synthesis, and optical properties of a new class of cyanines, **CyBX** NIR functional dyes. We further conducted quantum chemical calculations to shed light on the unique **CyBX** NIR functional dyes. Finally, to demonstrate the potential use of these innovative types of cyanines, we created two turn-on NIR fluorescent probes based on the **Spiro-CyBX** platform for imaging pH changes in living cells and Hg^2+^ in living animals.

## Results and discussion

### Design and synthesis of **CyBX** NIR fluorescent dyes

**Scheme 2:**
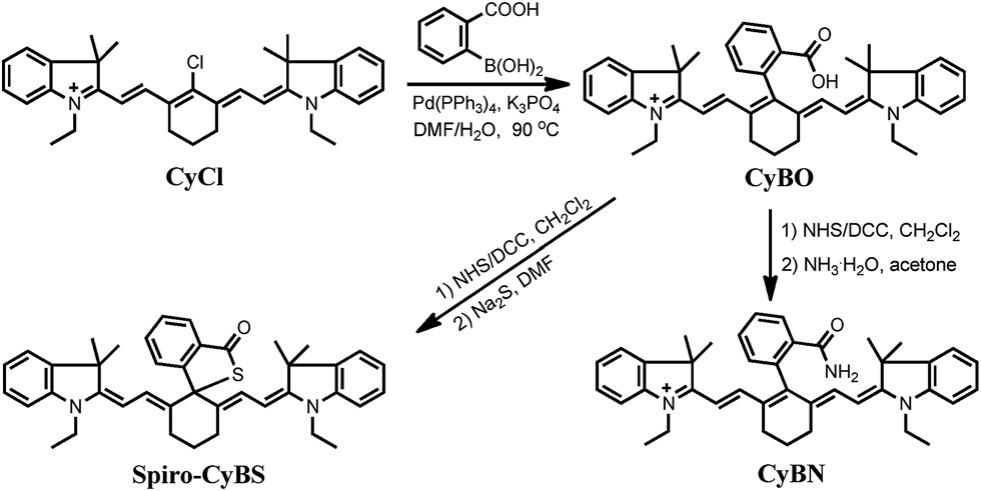
Synthesis of NIR functional **CyBX** dyes.

Heteroatoms such as O, N, and S have distinct nucleophilic natures. Thus, their cyclization abilities may significantly vary. Based on the above strategy, we anticipated that **CyBO**, **CyBN**, and **CyBS** compounds (Scheme 2) may have different extents of spiro-cyclization, which could confer diverse optical properties on these compounds. Thus, we expected that these dyes may be used for different situations in living systems.

The synthesis of the compounds **CyBO**, **CyBN**, and **CyBS** is shown in Scheme 2. The starting compounds CyCl and 2-carboxyphenylboric acid in DMF/H_2_O were heated under reflux in the presence of Pd(PPh_3_)_4_ to afford **CyBO** *via* the Suzuki–Miyaura method.^11^ This synthetic method is very simple with just one step. In addition, it is modular and versatile, as the carboxylic acid could be easily functionalized to afford its various derivatives. For instance, **CyBO** was firstly activated with a standard coupling reagent, *N*-hydroxysuccinimide (NHS), followed by reacting with ammonia or sodium sulfide to provide **CyBN** and **CyBS**, respectively. The structures of the **CyBX** products were fully characterized by ^1^H NMR, ^13^C NMR, MS-ESI, and HRMS (ESI†).

### Optical properties of **CyBX** NIR fluorescent dyes

The absorption and emission profiles of **CyBX** (**X** = **O**, **N**, or **S**) compounds in different organic solvents (DMF, DMSO, CH_3_COCH_3_, CH_2_Cl_2_, MeOH, EtOH, and CH_3_CN) are shown in Fig. S1–S3 (ESI†), and the corresponding photophysical data in DMF are compiled in Table S1 (ESI†). **CyBX** dyes show maximal absorption and emission at around 770 and 800 nm, respectively, which are well located in the NIR region. Notably, as designed, the shapes of the absorption/emission spectra of **CyBX** dyes highly resemble those of **CyCl** (Fig. S4, ESI†), which is in good agreement with the fact that **CyBX** dyes keep the same intact cyanine backbone as **CyCl** (Scheme 2). In addition, the photostability of **CyBX** dyes in DMF was measured by continuous irradiation with a Xe lamp (150 W) with a 10 nm slit width at the maximal absorption wavelength of the **CyBX** dyes. The results demonstrate that less than 6.4% of the initial fluorescence intensity was decreased after 1 hour of irradiation (Fig. S5†), indicating that these NIR dyes have sufficient photostability for potential biological imaging applications.

**Fig. 1:**
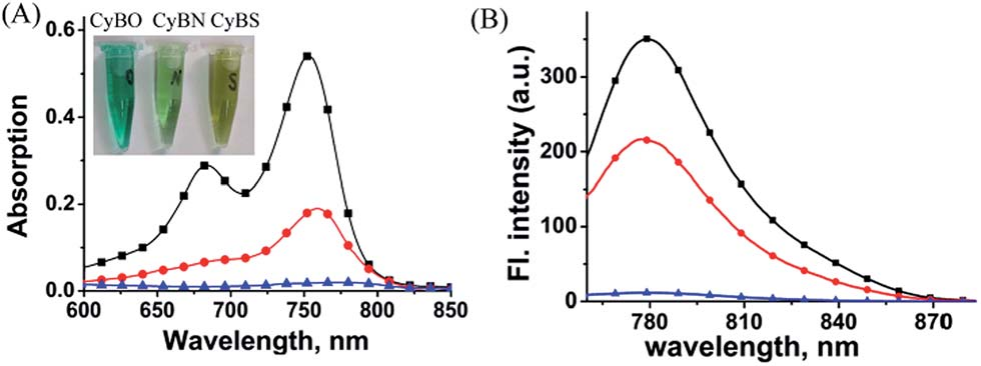
The absorption (A) and fluorescence (B) spectra of the compounds **CyBO** (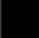), **CyBN** (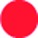), and **CyBS** (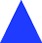) (10 μM) in pH = 7.4, PBS/DMF = 95/5. The excitation wavelengths are at 746 nm for **CyBO**, 745 nm for **CyBN**, and 748 nm for **CyBS**, respectively. Inset: (A) the visual colors of the **CyBX** dyes (left to right: **X** = **O**, **N**, and **S**) in PBS aqueous solution.

We then examined the optical properties of the **CyBX** dyes in aqueous solution. As expected, they showed drastic differences in absorption intensity in PBS buffer (pH 7.4, 5% DMF) (Fig. 1A). The compound **CyBO** exhibits strong absorption at around 770 nm, while **CyBN** has a much smaller absorption. By sharp contrast, **CyBS** displays almost no absorption. The visual colors of the **CyBX** dyes (0.3 mM, pH = 7.4, PBS/DMF, v/v = 1 : 1) are consistent with the absorption properties. **CyBO**, **CyBN**, and **CyBS** exhibit green, light green, and pale yellow colours, respectively (Inset of Fig. 1A). **CyBO** is highly fluorescent, while **CyBN** displays good fluorescence, and **CyBS** is essentially non-fluorescent (Fig. 1B). Thus, the fluorescence intensity of these dyes is in the order of **CyBO** > **CyBN** > **CyBS**, in accordance with the trend observed in the absorption. These results may be ascribed to the fact that the nucleophilic abilities of heteroatoms are in the order of S > N > O. Thus, the data suggest that in aqueous buffer; for **CyBO**, the opened-ring form (**CyBO**) is predominated; for **CyBN**, the opened-ring form (**CyBN**) and the spiro form (**Spiro-CyBN**) are in equilibrium; for **CyBS**, the spiro form (**Spiro-CyBS**) is essentially predominated.

**Fig. 2:**
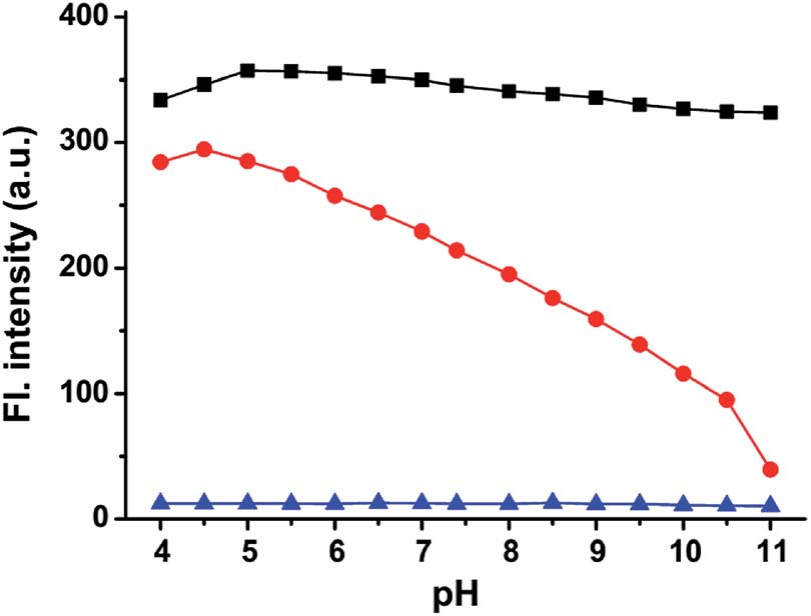
The maximum emission intensity of the compounds: **CyBO** (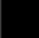), **CyBN** (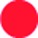), and **CyBS** (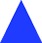) (10 μM, pH = 7.4, PBS/DMF = 95/5) at various pH values. The excitation wavelengths are at 746 nm for **CyBO**, 745 nm for **CyBN**, and 748 nm for **CyBS**, respectively.

To support the above hypothesis, we further investigated the fluorescence profiles of the new NIR dyes at different pH values. As shown in Fig. 2, **CyBO** shows strong fluorescence over a wide pH range of 4.0–11.0. It is worthy to note that, a gradual drop in the emission with increasing pH is consistent with the hypothesis that the spiro-cyclization ability of the carboxylic acid increases under basic conditions. In the situation of **CyBN**, a significant decrease in emission with increasing pH is observed. This can be explained by the equilibrium of the ring-opened form (**CyBN**) and the spiro form (**Spiro-CyBN**) shifting towards the spiro form (**Spiro-CyBN**) under basic conditions. In the case of **CyBS**, it essentially displays no fluorescence over a wide pH range of 4.0–11.0, implying that even under strong acidic conditions, the spiro form (**Spiro-CyBS**) is still predominated.

**Fig. 3:**
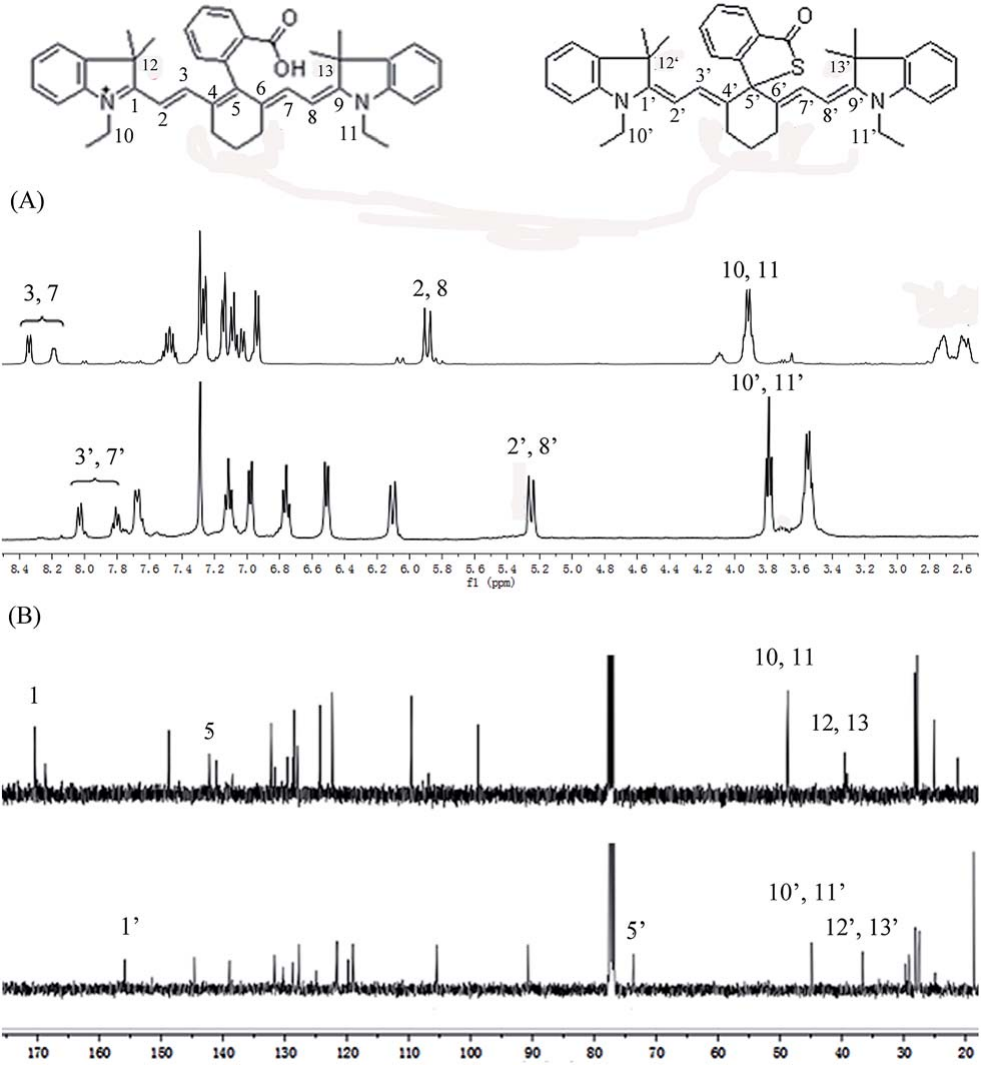
Comparison of partial ^1^H NMR (A) and ^13^C NMR (B) spectra of **CyBO** (top) and **CyBS** (bottom) in CDCl_3_.

To further confirm the design concept, we also compared both the ^1^H NMR and ^13^C NMR of **CyBO** and **CyBS**, as these two dyes have different predominant forms. In the case of **CyBO**, the ring-opened form (**CyBO**) is essentially predominated, while for **CyBS**, the spiro form (**Spiro-CyBS**) is predominated. Thus, we should observe a pronounced distinction between **CyBO** and **CyBS** in the NMR spectra. Indeed, the chemical shifts of the protons of **CyBS** exhibited a marked shift in respect to those of **CyBO** (Fig. 3A). This is consistent with the hypothesis that the spiro-cyclization of **CyBS** leads to the disappearance of the positive charge on the nitrogen, which should confer a strong effect on the chemical shifts of the protons. Importantly, the presence of **CyBS** in the spiro form and **CyBO** in the ring-opened form is clearly evident in ^13^C NMR spectra. As shown in Fig. 3B, the key 5-position carbon in **CyBO** displays a chemical shift at around 140 ppm, in good agreement with its sp^2^ character. By sharp contrast, the corresponding 5′-position carbon in **CyBS** has a chemical shift at around 73 ppm, in accordance with its sp^3^ character due to the spiro-cyclization.

### Density functional theory (DFT) calculations

**Fig. 4:**
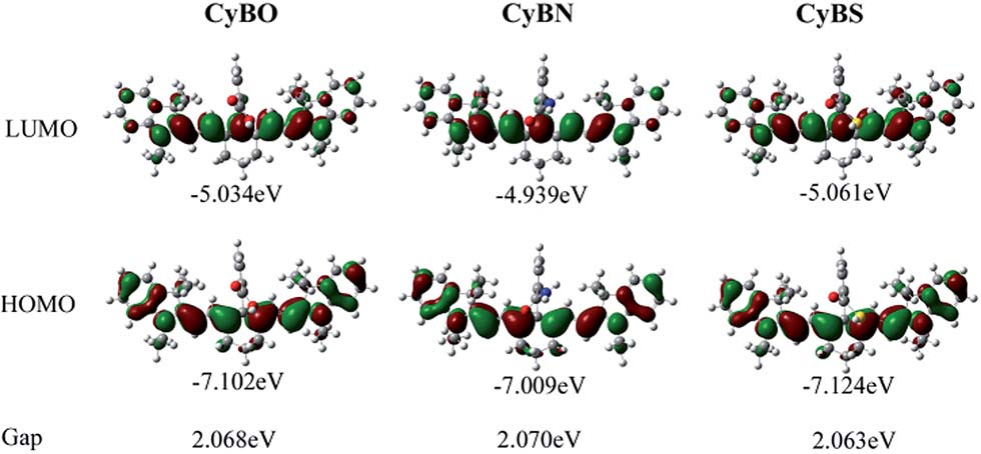
Molecular orbital plots (LUMO and HOMO) and HOMO–LUMO energy gaps of NIR **CyBX** (**X** = **O**, **N**, or **S**) dyes.

To shed light on the optical properties of the new functional NIR fluorescent **CyBX** dyes, DFT calculations with the B3LYP exchange functional employing 6-31G* basis sets using a suite of Gaussian 09 programs were conducted.^12^ The frontier molecular orbital plots of the **CyBX** dyes are shown in Fig. 4. The HOMO–LUMO energy gaps of the dyes are very close, consistent with the above findings that these dyes have similar maximal absorption wavelengths (Table S1, ESI†). Furthermore, the DFT optimized structures of the **CyBX** dyes reveal that the benzoic acid (benzamide, or benzothioic acid) moiety is nearly perpendicular to the heptamethine cyanine core (Fig. S6 and Table S2, ESI†), indicating that the benzoic acid (benzamide, or benzothioic acid) unit at the *meso*-position has a very minor contribution to the π–π conjugated backbone. This is in good agreement with the observation that the benzoic acid (benzamide or benzothioic acid) unit has almost no contribution to the HOMO–LUMO (Fig. 6). We then calculated the charge distribution of the optimized structures of the **CyBX** dyes by natural bond orbital (NBO) analysis at the B3LYP level (Fig. S7, ESI†). The representative atomic charges are displayed in Table S3 (ESI†). 1-, 9-position (C=N) and 5-position (central) carbons exhibit a positive charge, while all other carbons on the conjugated backbone show a negative charge. However, considering the three-dimensional structures of the **CyBX** dyes, only the central (5-position) carbon is likely to be attacked by the nucleophilic heteroatoms (O, N, or S) to form the five-membered spiro form (**Spiro-CyBX**).

### Development of an innovative NIR fluorescent pH probe for biological imaging

The above studies indicate that, like heptamethine cyanine, the new functional NIR **CyBX** dyes have both absorption and emission in the NIR region. Furthermore, importantly, **CyBX** dyes have an intrinsic fluorescence switch by spiro-cyclization. These highly favorable characters imply that **CyBX** dyes could act as robust platforms for design of NIR fluorescent probes. In addition, the standard MTT assays indicate that **CyBX** dyes have negligible cytotoxicity to living cells (Fig. S8, ESI†), suggesting that the new compounds are promising for applications in biological systems. In addition, to investigate the cellular uptake of **CyBX** dyes, we chose **CyBO** as a representative dye for the time-dependent confocal fluorescence imaging of living cells, up to 120 min (Fig. S9, ESI†). Efficient cellular uptake was observed in the first 30 min of incubation, while the fluorescence was not significantly different after incubating for 30, 60, and 120 min. These data suggest that the cellular uptake of the dye might be *via* endocytosis.^13^

**Scheme 3:**
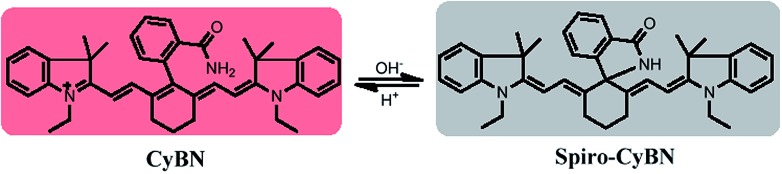
The ring-opened and spiro form of the NIR probe **CyBN** under acidic and basic conditions.

Toward this end, we first tested the possibility of **CyBN** as a novel NIR fluorescent pH probe, as it is sensitive to pH variations as aforementioned (Fig. 2). Intracellular pH (pH_i_) plays an important role in many biological events including cell proliferation and apoptosis,^14^ enzymatic activity,^15^ and ion transport.^16^ In a typical mammalian cell, the pH_i_ can vary from 4.7 in lysosomes to 8.0 in mitochondria.^17^ However, abnormal intracellular pH variations are associated with diseases such as Alzheimer's disease and cancer.^18^ Therefore, monitoring pH changes inside living cells is critical for investigating both physiological and pathological processes. So far, a diverse array of fluorescent pH probes have been developed.^8e–g,19^ However, only very few of them have both absorption and emission in the NIR region. In addition, most of the reported NIR fluorescent pH probes function by a PET mechanism. By contrast, herein we exploited **CyBN** as a candidate NIR fluorescent pH probe aiming to demonstrate that the internal fluorescence switch by spiro-cyclization in cyanines could effectively be employed for NIR pH probe development (Scheme 3).

**Fig. 5:**
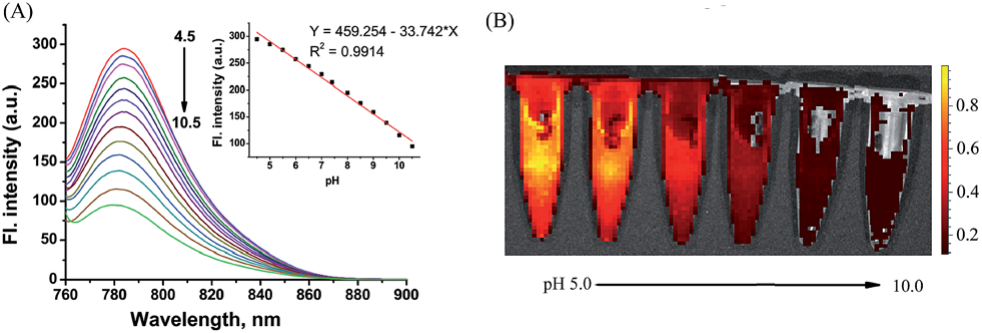
(A) pH-dependence of the fluorescence spectra of the NIR probe **CyBN** (10 μM, PBS/DMF: 95/5) upon excitation at 745 nm. Inset: plot of the fluorescence intensity *versus* pH. (B) The fluorescent images of **CyBN** (10 μM, DMF/PBS 5/95) at pH 5.0 to 10.0 using the IVIS Lumina XR (IS1241N6071) *in vivo* imaging system with an excitation filter of 675 nm and an emission range of 760–810 nm.

As shown in Fig. 5A, **CyBN** (10 μM, in PBS aqueous containing 5% DMF) displays strong fluorescence at pH 4.5. However, an increase in pH induces a gradual decrease of emission, in good agreement with the fluorescence images recorded by an *in vivo* imaging system (IVIS Lumina XR (IS1241N6071)) (Fig. 5B). The pH-dependence changes in the emission can be rationalized as the fluorescent ring-opened form of **CyBN** dominates under acidic conditions but the non-fluorescent spiro form dominates under basic conditions. The variations in the absorption profiles further support this explanation (Fig. S10, ESI†).

**Fig. 6:**
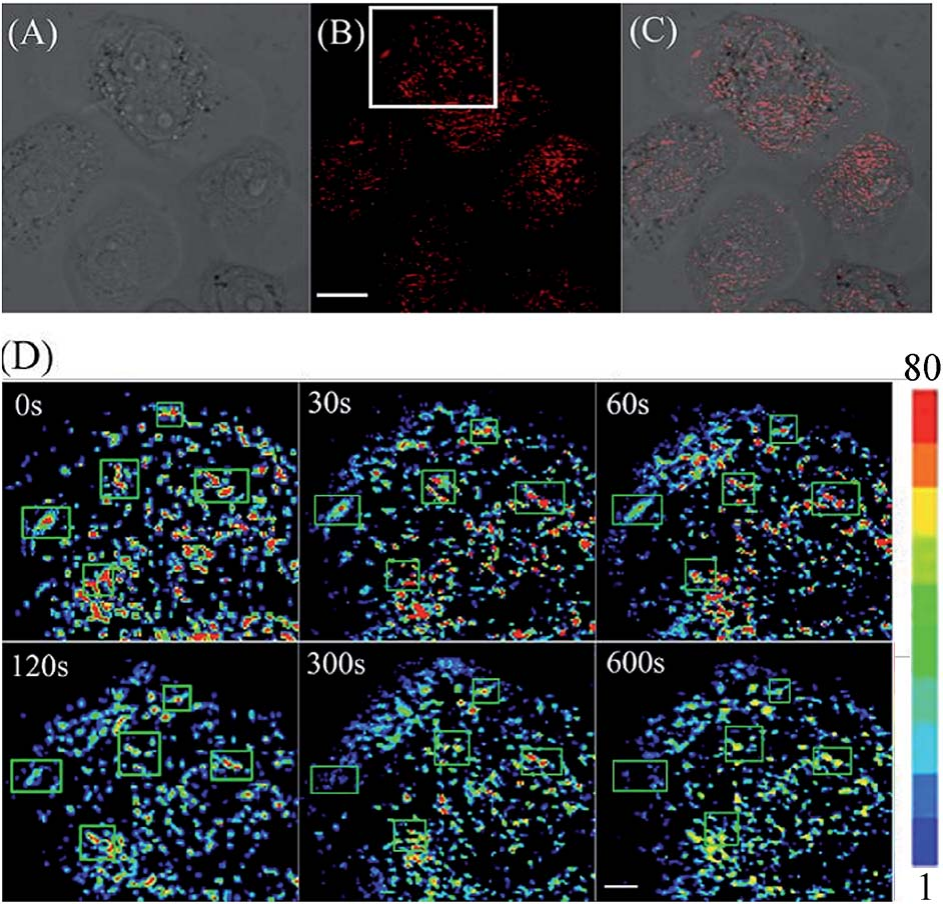
(A) Brightfield image of EC109 cells stained with **CyBN** (5 μM) in pH 7.0 PBS solution. (B) Confocal fluorescence image of (A). (C) Overlay of (A) and (B). (D) Followed by addition of 10 mM NH_4_Cl, and then image (B) was continuously imaged within 10 min. The white box in (B) was enlarged and has a pseudocolor showing the changes of pH with time. The changes of pH were highlighted by green boxes. All the images of fluorescence emission were collected between 770 to 810 nm upon excitation at 635 nm. Scale bar = 10 μm for (A–C) and 5 μm for (D).

Encouraged by these results, we decided to employ **CyBN** to monitor real-time changes of pH in living cells. Toward this end, EC109 cells were incubated with the probe (5 μM) at 37 °C for 30 min, and then the cells were washed with pH 7.0 PBS medium (3 × 1 mL). Upon addition of NH_4_Cl, a weak basic reagent that can be used to increase intracellular pH,^20^ the variations of the fluorescence intensity of the cells were continuously recorded within 10 minutes (Fig. 6 and S11, ESI†). The results indicate that the dye-stained cells display a real-time drop in emission upon the addition of NH_4_Cl, which is in good agreement with the pH-dependent changes in the fluorescence spectra of **CyBN** in aqueous solution (Fig. 5A).

**Fig. 7:**
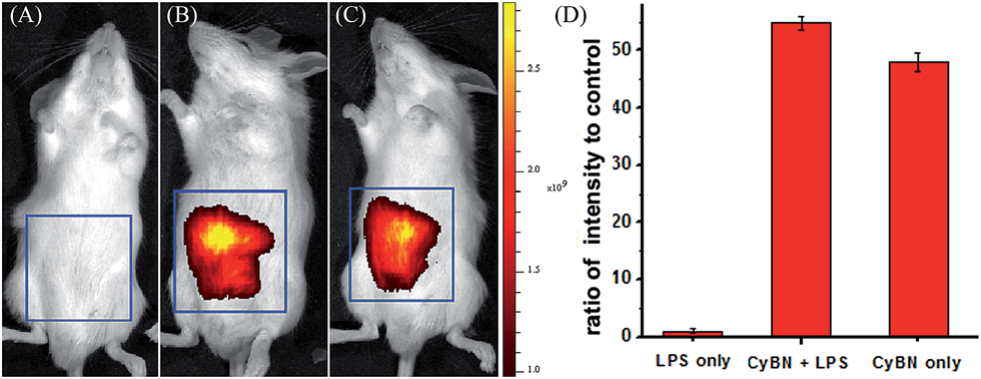
Representative fluorescence images (pseudocolor) of the mouse injected with **CyBN** during LPS-mediated inflammatory response *in vivo*. (A) Only LPS was injected as a control. (B) LPS was injected into the peritoneal cavity of the mice, followed by an injection with **CyBN** (50 nmol). (C) Only **CyBN** (50 nmol) was injected. (D) Quantification of the emission intensity from the abdominal area of the mice of the experimental groups relative to the control group. All mice were imaged using the IVIS Lumina XR (IS1241N6071) *in vivo* imaging system with an excitation filter of 745 nm and an emission range of 760–810 nm.

We further investigated the possibility of **CyBN** in visualizing endogenous pH changes in an abdominal inflammation model induced by lipopolysaccharides (LPS).^21^ Two Kunming mice were intraperitoneally (i.p.) injected with LPS to produce an acute inflammatory response, and one of the mice was then injected with **CyBN**. As shown as in Fig. 7, the emission of the mouse loaded with both LPS and **CyBN** is stronger than that of the mouse incubated with only the probe **CyBN**. As a control, the mouse treated with only LPS exhibited almost no fluorescence (Fig. 7a). The fluorescence intensity from the abdominal area of the mice was quantified, and the inflamed mouse treated with **CyBN** exhibited a higher fluorescence intensity than the normal mouse (Fig. 7D), which suggests a decrease of the pH value in the inflammatory tissues, which is consistent with a previous report.^22^ Taken together, these results demonstrate that **CyBN** is useful for *in vivo* imaging and can detect the pH changes in small animals.

### Development of a new NIR fluorescent turn-on Hg^2+^ probe for biological imaging in living animals

**Scheme 4:**
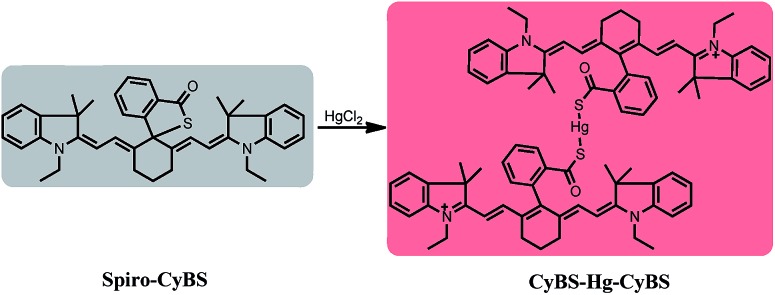
The likely sensing mechanism of **CyBS** with Hg^2+^.

The thiol-affinity to Hg^2+^ has been exploited to construct fluorescent probes for mercury cations.^23^ **CyBS** contains a strong nucleophilic benzothioate and shows almost no fluorescence as the spiro form (**Spiro-CyBS**) dominates. Thus, we envisioned that, upon reacting with Hg^2+^, the thiol moiety of non-fluorescent **Spiro-CyBS** may coordinate with Hg^2+^ resulting in the formation of highly fluorescent **CyBS-Hg-CyBS** (Scheme 4). In other words, **CyBS** may exhibit a fluorescence-enhanced signal in the presence of Hg^2+^.

**Fig. 8:**
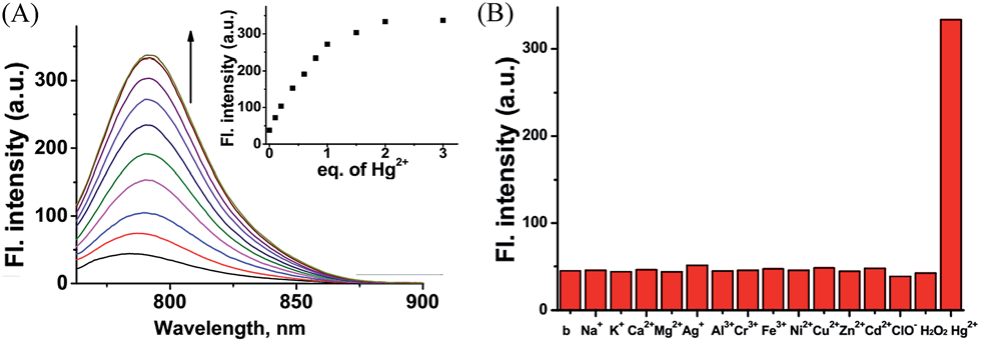
(A) Fluorescence spectra of **CyBS** (10 μM) in the presence of various concentrations of Hg^2+^ (0–30 μM) in phosphate buffer (pH 7.4, 30% MeOH) with excitation at 745 nm. Inset: fluorescence intensity of **CyBS** at 745 nm *vs.* Hg^2+^ concentration (0–3 equivalents). (B) Fluorescence intensity at 792 nm of **CyBS** (10 μM) with excitation at 745 nm in the presence of various species, such as 200 equivalents of K^+^, Na^+^, Ca^2+^, and Mg^2+^; 20 equivalents of Ag^+^, Al^3+^, Cr^3+^, Fe^3+^, Ni^2+^, Cu^2+^, Zn^2+^, and Cd^2+^; 2 equivalents of ClO^–^, H_2_O_2_, and Hg^2+^.

As designed, the free probe **CyBS** is almost non-fluorescent in PBS solution (pH 7.4, 30% MeOH). However, addition of Hg^2+^ ions elicits a dramatic effect on both the fluorescence and absorption spectra (Fig. 8A and S12, ESI†). A significant fluorescence turn-on response is observed due to the Hg^2+^-mediated formation of the opened form, and the detection limit was calculated to be 7.27 × 10^–7^ M (*S*/*N* = 3) (Fig. S13, ESI†). Mass spectrometry analysis confirms that the probe coordinates with Hg^2+^ to form a fluorescent ring-opened form of **CyBS-Hg-CyBS** (Fig. S14, ESI†), which is consistent with the sensing mechanism reported for benzothioate-based fluorescent Hg^2+^ probes.^23^ Furthermore, as displayed in Fig. 8B, the probe is highly selective for Hg^2+^ over other various relevant metal ions (K^+^, Na^+^, Ca^2+^, Mg^2+^, Ag^+^, Al^3+^, Cr^3+^, Fe^3+^, Ni^2+^, Cu^2+^, Zn^2+^, and Cd^2+^) and reactive oxygen species (ROS) including HClO and H_2_O_2_.

**Fig. 9:**
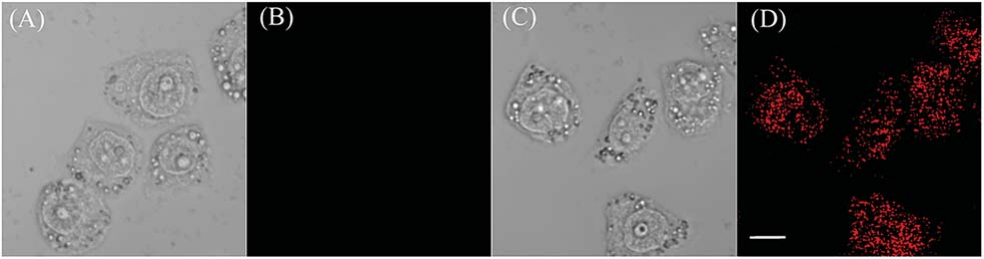
Images of EC109 cells treated with the probe **CyBS** in the absence or presence of Hg^2+^. (A) Brightfield image of EC109 cells incubated with only **CyBS** (5 μM) for 30 min; (B) fluorescence image of (A); (C) brightfield image of EC109 cells incubated with **CyBS** (5 μM) for 30 min and further treated with Hg^2+^ (5 μM) for another 5 min; and (D) fluorescence image of (C). Fluorescence images (B) and (D) were collected between 770 to 810 nm upon excitation at 635 nm. Scale bar = 10 μm.

We proceeded to evaluate the ability of the probe to function in living cells. EC109 cells incubated with **CyBS** (5 μM) for 30 min at 37 °C provide almost no fluorescence (Fig. 9B). By contrast, the cells incubated with the probe and then Hg^2+^ had a strong fluorescence (Fig. 9D). These results demonstrate that **CyBS** is cell membrane permeable and can respond to Hg^2+^ with a turn-on signal in the living cells.

**Fig. 10:**
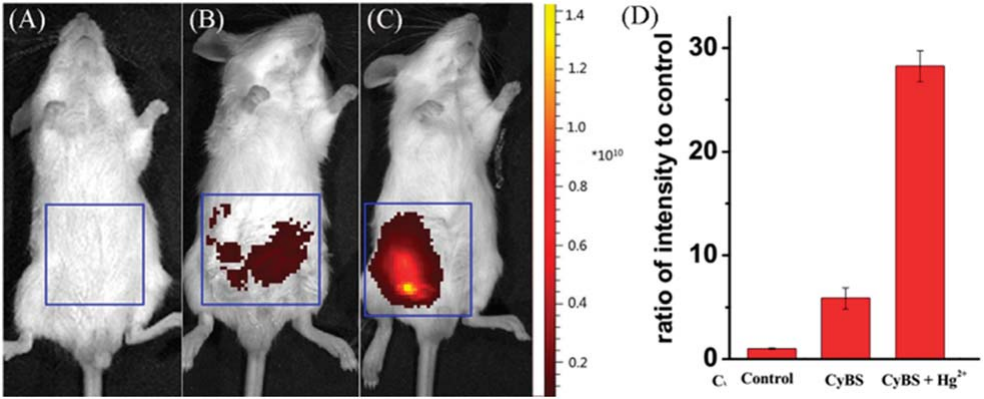
Representative fluorescence images (pseudocolor) of the mice. (A) Fluorescence image of the negative control, neither **CyBS** nor Hg^2+^ was injected; (B) fluorescence image of the mouse treated with only **CyBS** (50 nmol) for 10 min; (C) fluorescence image of the mouse injected with **CyBS** (50 nmol) for 10 min, followed by an i.p. injection of Hg^2+^ (100 nmol); (D) quantification of the emission intensity from the abdominal area of the mice of the experimental groups relative to the control group. All mice were imaged using the IVIS Lumina XR (IS1241N6071) *in vivo* imaging system with an excitation filter of 745 nm and an emission range of 760–810 nm.

To further demonstrate the potential of the probe for imaging applications in living animals owing to its advantageous NIR absorption and emission, the probe was given to mice in the absence or presence of Hg^2+^ ions. As shown in Fig. 10B, the control Kunming mouse i.p. injected with only **CyBS** (50 nmol) had very weak fluorescence. However, followed by i.p. injection with Hg^2+^ (100 nmol) at the same site, a significant increase of fluorescence intensity was noted (Fig. 10C and D). These results are in good agreement with the data aforementioned in aqueous solution (Fig. 8A) and living cells (Fig. 9). Taken together, these studies indicate that the new NIR probe **CyBS** can image Hg^2+^ not only in living cells but also in living animals, demonstrating its value.

## Conclusions

In summary, we introduced a simple and effective capping approach to readily tune the fluorescence of NIR cyanines. The unique strategy is based on direct installation of a benzoic acid (benzamide, or benzothioic acid) moiety onto the intact cyanine backbone. The resulting new functional NIR **CyBX** (**X** = **O**, **N**, or **S**) dyes not only retain the intact tricarbocyanine scaffold, but also have a built-in switch to regulate fluorescence by spiro-cyclization. Thus, like traditional NIR cyanines, **CyBX** dyes exhibit both absorption and emission in the NIR region. Furthermore, importantly, **CyBX** dyes display a unique nature; their NIR optical properties can be readily tuned by the intrinsic spiro-cyclization mechanism. To our best knowledge, such types of NIR cyanines is unprecedented. We further showed that **CyBN** could be employed to monitor real-time pH changes in living systems and **CyBS** could be used to detect Hg^2+^ in both living cells and living animals with a turn-on signal, demonstrating the values of the NIR functional fluorescent **CyBX** dyes. We expect that the capping strategy can be extended across not only the visual spectrum and but also to structurally distinct fluorophore species. More broadly, the findings described herein suggest the possible development of a series of next-generation functional dyes with optical profiles that are readily tunable. A diverse array of optical probes with such exciting properties would likely find widespread application as powerful molecular tools in studies involving fluorescence microscopy.

## Ethical statement

Kunming mice were purchased from Experimental Animal Center of Xiangya School of Medicine Central South University (Changsha, China). All animal procedures for this study were approved by the Animal Ethical Experimentation Committee of Central South University according to the requirements of the National Act on the use of experimental animals (China).
