# Supplementary material for: A simple and effective “capping” approach to readily tune the fluorescence of near-infrared cyanines
Source: Chem Sci. 2015 May 5;6(8):4530–6. doi: 10.1039/c5sc00348b (PMC5499489; doi:10.1039/c5sc00348b)
Supplement: Supplementary file 1 [file SC-006-C5SC00348B-s001.pdf]

Electronic Supplementary Material (ESI) for Chemical Science.
This journal is © The Royal Society of Chemistry 2015

Supplementary information for

A Simple and Effective "Capping" Approach to Readily Tune the Fluorescence of Near-infrared Cyanines

Longwei He,[†] Weiying Lin,*[‡,†] Qiuyan Xu,[†] Mingguang Ren,[‡] Haipeng Wei,[†] and Jian-Yong Wang[‡]

[‡] Institute of Fluorescent Probes for Biological Imaging, School of Chemistry and Chemical Engineering, School of Biological Science and Technology, University of Jinan, Jinan, Shandong 250022, P.R. China.

[†] State Key Laboratory of Chemo/Biosensing and Chemometrics, College of Chemistry and Chemical Engineering, Hunan University, Changsha, Hunan 410082, P. R. China.

Email: weiyinglin2013@163.com

Table of contents

Page

Materials and instruments…………. ………………………………… …….S3

Determination of the fluorescence quantum yield…………………… ……….S3

Synthesis……………………… ……………………………………..S4-5

Preparation of Cell Cultures……….. ……………………………….………..S5

Imaging of pH Changes in Living Cells Using CyBN…………………..……..S5

Imaging of pH Changes in Living Mice Using CyBN…………………..……..S6

Imaging of Hg^{2+} in Living Cells Using CyBS…………………………..……..S6

Imaging of Hg^{2+} in Living Mice Using CyBS……………..…………………..S6

Figure S1-3……………………… ………………………………………S7

Figure S4……………………………………………………………………..S8

Table S1…………………………… ……….. ……………………………S8

Figure S5-6…………………………………………………………………..S9

Table S2…………………………… ……….. ……………………..….S10

Figure S7……………………………………………………………….....S10

Table S3…………………………… ……….. …………………………...S11

Figure S8……………………………………………………………………S11

Figure S9-10..……………………………………………………..…..S12

Figure S11-12………………………………………………………………S13

Figure S13-14………………………………………………………………S14

Spectral Characterization……………………..…………………………S15-17

Materials and instruments: Unless otherwise stated, all reagents were purchased from commercial suppliers and used without further purification. Solvents used were purified by standard methods prior to use. Twice-distilled water was used throughout all experiments; Low resolution mass spectra were performed using an LCQ Advantage ion trap mass spectrometer from Thermo Finnigan or Agilent 1100 HPLC/MSD spectrometer; High-resolution electronspray (ESI-HRMS) mass spectra were obtained from Bruker APEX IV-FTMS 7.0T mass spectrometer; NMR spectra were recorded on an INOVA-400 spectrometer, using TMS as an internal standard; Electronic absorption spectra were obtained on a LabTech UV Power spectrometer; Photoluminescent spectra were recorded with a HITACHI F4600 fluorescence spectrophotometer with a 1 cm standard quartz cell; The fluorescence imaging of cells was performed with OLYMPUS FV1000 (TY1318) confocal microscopy; The fluorescence imaging of mice and solution was performed with IVIS Lumina XR (IS1241N6071) *in vivo* imaging system; The pH measurements were carried out on a Mettler-Toledo Delta 320 pH meter; TLC analysis was performed on silica gel plates and column chromatography was conducted over silica gel (mesh 200–300), both of which were obtained from the Qingdao Ocean Chemicals.

Determination of the fluorescence quantum yield: Fluorescence quantum yields for **CyBX** were determined by using ICG (Φ_f = 0.13 in DMSO) as fluorescence standard.[1] The quantum yield was calculated using the following equation (1):

$$\Phi_{F(X)} = \Phi_{F(S)} (A_S F_X / A_X F_S) (n_X / n_S)^2 \qquad (1)$$

Where Φ_F is the fluorescence quantum yield, A is the absorbance at the excitation wavelength, F is the area under the corrected emission curve, and n is the refractive index of the solvent used. Subscripts $_S$ and $_X$ refer to the standard and to the unknown, respectively.

1. (a) Valeur, B. *Molecular Fluorescence: Principles and Applications*, Wiley-VCH, 2001; (b) Oushiki, D.; Kojima, H.; Terai,T.; Arita, M.; Hanaoka, K.; Urano, Y.; Nagano. T. *J. Am. Chem. Soc.* **2010**, *132*, 2795-2801.

Synthesis of CyBO. Cy-Cl (63.8 mg, 0.1 mmol), 2-carboxyphenylboric acid (33.2 mg, 0.2 mmol), and $K_3PO_4 \cdot 7H_2O$ (33.8 mg, 0.1 mmol) dissolved in 3 mL DMF/H_2O (v/v = 5:1) were heated at 90 °C in the presence of $Pd(PPh_3)_4$ (11.6 mg, 0.01 mmol) for 5 h. The reaction mixture was cooled to room temperature, and then 100 mL ice water was added. The mixture was extracted three times with CH_2Cl_2/CH_3CH_2OH (v/v = 10:1). The organic phase was separated, washed with brine, and dried with anhydrous $MgSO_4$. The solvent was removed under reduced pressure to give the crude product, which was purified by silica gel flash chromatography using CH_2Cl_2 to CH_2Cl_2/CH_3CH_2OH (v/v 100:1 to 5:1) as eluent to afford the compound **CyBO** as a green solid (22 mg, 30.4% yield). ^1H NMR (400 MHz, $CDCl_3$): 1.11 (s, 6H), 1.18 (s, 6H), 1.30 (6H), 1.95-2.03 (m, 2H), 2.56-2.60 (2H), 2.71-2.75 (2H), 3.89-3.94 (q, *J* = 7.2, 4H), 5.87-5.91 (d, *J* = 14.0, 2H), 6.93-6.95 (d, *J* = 7.6, 2H), 7.02-7.04 (d, *J* = 7.2, 1H), 7.06-7.10 (2H), 7.14-7.15 (2H), 7.26-7.30 (3H), 7.46-7.50 (2H), 8.18-8.20 (d, *J* = 4.8, 1H), 8.33-8.35 (d, *J* = 7.2, 1H). ^{13}C NMR (100 MHz, $CDCl_3$): 11.9, 21.0, 24.8, 27.6, 28.0, 39.3, 48.5, 98.6, 106.6, 109.3, 122.1, 124.1, 127.8, 128.3, 128.5, 129.4, 131.4, 132.0, 138.2, 140.9, 142.0, 148.5, 168.5, 170.2. MS (ESI) *m/z* 597.3 $[M-I]^+$. HRMS (ESI) *m/z* calcd for $C_{41}H_{45}N_2O_2^+$ (M^+): 597.3476. Found 597.3451.

Synthesis of CyBN and CyBS. CyBO (14.5 mg, 0.02 mmol), N-Hydroxysuccinimide (3.5 mg, 0.03 mmol), and N,N'-Dicyclohexyl carbodiimide (8.2 mg, 0.04 mmol) were dissolved in 3 mL dichloromethane, and the reaction mixture was stirred at room temperature for 1 h. The solvent was removed under reduced pressure to give the intermediate, which was dissolved in 3 mL acetone in the presence of 0.5 mL concentrated ammonia to react another 5 h at room temperature. The reaction mixture was concentrated under reduced pressure to give the crude product, which was purified by silica gel flash chromatography using CH_2Cl_2 to CH_2Cl_2/CH_3CH_2OH (v/v 100:1 to 10:1) as eluent to afford the compound **CyBN** as a light green solid (9.2 mg, 63.8% yield).

For the synthesis of **CyBS**, the intermediate was dissolved in DMF (3 mL) and further reacted with Na_2S (2 equivalents) for 0.5 h at room temperature. The reaction mixture was added into 50 mL water and extracted three times with CH_2Cl_2. The organic phase was separated, washed with brine, and dried with anhydrous $MgSO_4$. The solvent was removed under reduced pressure to give the crude product, which was purified by silica gel flash chromatography using CH_2Cl_2 to CH_2Cl_2/CH_3CH_2OH (v/v 50:1) as eluent to afford the compound **CyBS** as a yellow solid (6.3 mg, 42.6% yield).

CyBN: ^1H NMR (400 MHz) in $CDCl_3$: 1.24 (s, 6H), 1.26 (s, 12H) 1.36-1.39 (t, *J* = 7.2, 6H), 2.09-2.14 (2H), 2.47-2.53 (2H), 2.75-2.79 (2H), 4.03-4.08 (t, *J* = 7.2, 4H), 6.03-6.07 (d, *J* = 14.0, 2H), 7.04-7.06 (d, *J* = 8.0, 2H), 7.15-7.18 (3H), 7.21-7.22 (3H), 7.32 (s, 1H), 7.34-7.35 (2H), 7.53-7.55 (1H), 7.63-7.65 (2H); ^{13}C NMR (100 MHz) in $CDCl_3$: 12.1, 21.2, 24.8, 27.3, 27.7, 39.4, 48.9, 99.8, 110.2, 122.1, 124.9, 126.6, 128.6, 129.1, 130.2, 131.3, 134.7, 141.0, 141.8, 148.0, 171.4. MS (ESI) *m/z* 596.3 $[M-I]^+$. HRMS (ESI) *m/z* calcd for $C_{41}H_{46}N_3O^+$ (M^+): 596.3635. Found 596.3640.

CyBS: ^{1}H NMR (400 MHz, $CDCl_3$): 1.17-1.21 (12H), 1.26 (6H), 1.93-1.99 (m, 2H), 3.54-3.59 (q, J = 6.8 Hz, 4H), 3.79-3.82 (t, *J* = 6.4, 4H), 5.24-5.27 (d, *J* = 12.0, 2H), 6.08-6.11 (d, *J* = 11.6, 2H), 6.49-6.50 (d, *J* = 7.6, 2H), 6.72-6.76 (t, J = 7.2, 2H), 6.95-6.97 (d, *J* = 7.2, 2H), 7.07-7.11 (t, *J* = 7.6, 2H), 7.63-7.66 (2H), 7.76-7.79 (2H), 7.99-8.01 (d, *J* = 7.26, 1H). ^{13}C NMR (100 MHz, $CDCl_3$): 11.0, 18.7, 24.9, 27.4, 27.6, 28.1, 29.1, 29.1, 29.7, 36.6, 44.8, 73.5, 73.6, 90.6, 105.3, 118.8, 119.6, 121.4, 124.8, 127.6, 128.5, 130.1, 131.5, 138.7, 144.4, 155.6. MS (ESI) *m/z* 613.2 $[M-I]^+$. HRMS (ESI) *m/z* calcd for $C_{41}H_{45}NO^+$ (M^+): 613.3247. Found 613.3226.

Preparation of Cell Cultures. EC109 cells were maintained in DMEM (Dulbecco's modified Eagle's medium) supplemented with 10% FBS (fetal bovine serum) containing minimum essential medium (MEM), penicillin, streptomycin, and bovine insulin (0.01 mg/mL). Cells were grown in an atmosphere of 5% CO_2 and 95% air at 37 °C.

Imaging of pH Changes in Living Cells Using CyBN. EC109 cells were incubated with **CyBN** (5 μM) for 30 min in an atmosphere of 5% CO_2 and 95% air, followed by washing with pH 7.0 PBS medium three times and were incubated in 0.5 mL pH 7.0 PBS medium for another 10 min. The cells were then imaged using OLYMPUS FV1000 (TY1318) confocal microscopy with an excitation filter of 635 nm and an emission range of 770-810 nm. Followed by addition of 10 μL 0.5 M NH_4Cl (final concentration of 10 mM) into the medium, the cells were then continuously imaged within next 10 min.

Imaging of pH Changes in Living Mice Using CyBN. The Kunming mice were divided into three groups. The mice of the first group were anesthetized and then injected with LPS (1 mg in 400 μl saline) in the peritoneal cavity. The mice of the second group injected with **CyBN** (50 nmol in 100 μL CH_3OH) in the peritoneal cavity under anesthetic. For the third group, the anesthetic mice were first intraperitoneally injected with LPS (1 mg in 400 μl saline) in the peritoneal cavity, after 4 hours, followed by an intraperitoneal injection of **CyBN** (50 nmol in 100 μL CH_3OH) at the same site. The mice were imaged using IVIS Lumina XR (IS1241N6071) *in vivo* imaging system with an excitation filter of 745 nm and an

emission range of 760-810 nm.

Imaging of Hg^{2+} in Living Cells Using CyBS. EC109 cells were incubated with **CyBS** (5 μM) for 30 min in an atmosphere of 5% CO_2 and 95% air, and washed tree times with PBS medium, followed by addition of Hg^{2+} (5 μM) and incubated for another 5 min. The cells were imaged using OLYMPUS FV1000 (TY1318) confocal microscopy with an excitation filter of 635 nm and an emission range of 770-810 nm.

Imaging of Hg^{2+} in Living Mice Using CyBS. The Kunming mice were divided into three groups. The mice of the first group were anesthetized and then injected with saline (0.2 mL) in the peritoneal cavity. The mice of the second group injected with **CyBS** (50 nmol in 100 μL CH_3OH) in the peritoneal cavity under anesthetic. For the third group, the anesthetic mice were first intraperitoneally injected with **CyBS** (50 nmol in 100 μL CH_3OH) in the peritoneal cavity, after 10 min, followed by an intraperitoneal injection of Hg^{2+} (100 nmol in 100 μL deionized water) at the same site. The mice were imaged using IVIS Lumina XR (IS1241N6071) *in vivo* imaging system with an excitation filter of 745 nm and an emission range of 760-810 nm.

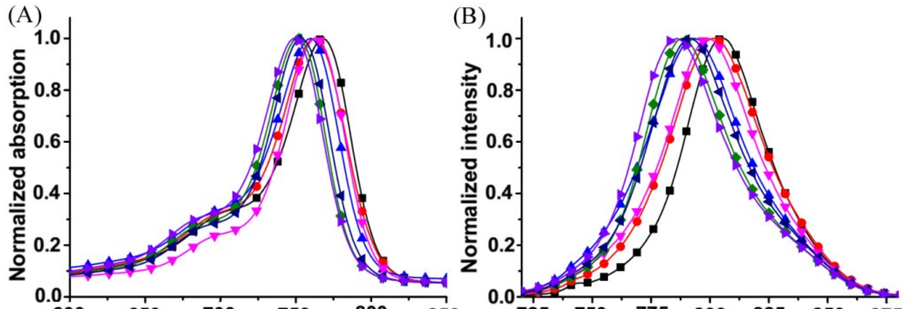

Figure S1. The absorption (A) and fluorescence (B) spectra of compound **CyBO** in

distinct organic solvents, including DMF (■), DMSO (●), acetone (▲), DCM (▼), MeOH (◆), EtOH (◀), and MeCN (▶).

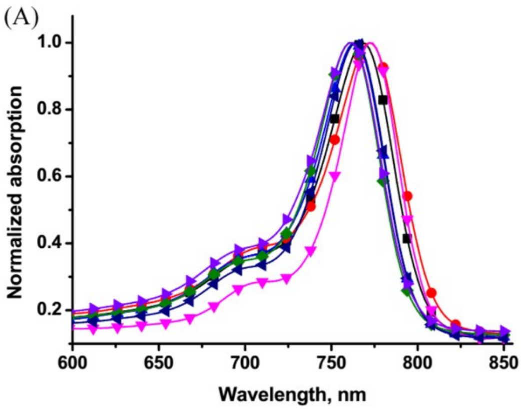

Figure S2. The absorption (A) and fluorescence (B) spectra of compound **CyBN** in distinct organic solvents, including DMF (■), DMSO (●), acetone (▲), DCM (▼), MeOH (◆), EtOH (◀), and MeCN (▶).

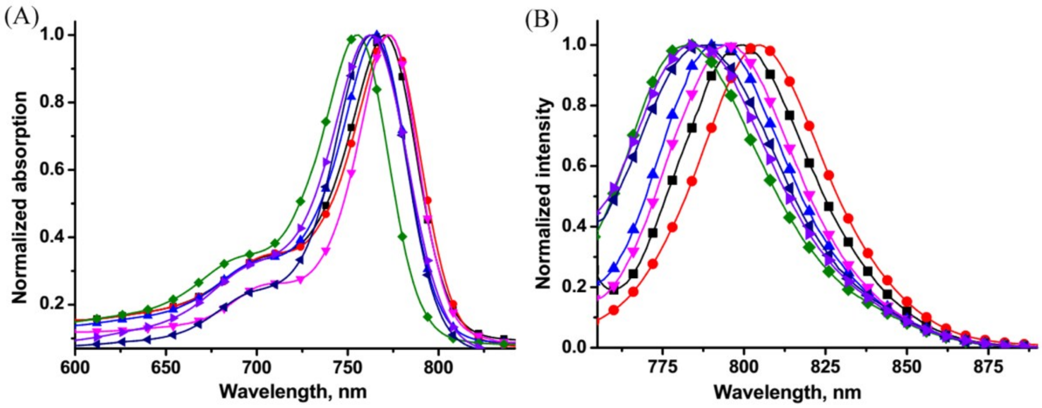

Figure S2. The absorption (A) and fluorescence (B) spectra of compound **CyBO** in distinct organic solvents, including DMF (■), DMSO (●), acetone (▲), DCM (▼), MeOH (◆), EtOH (◀), and MeCN (▶).

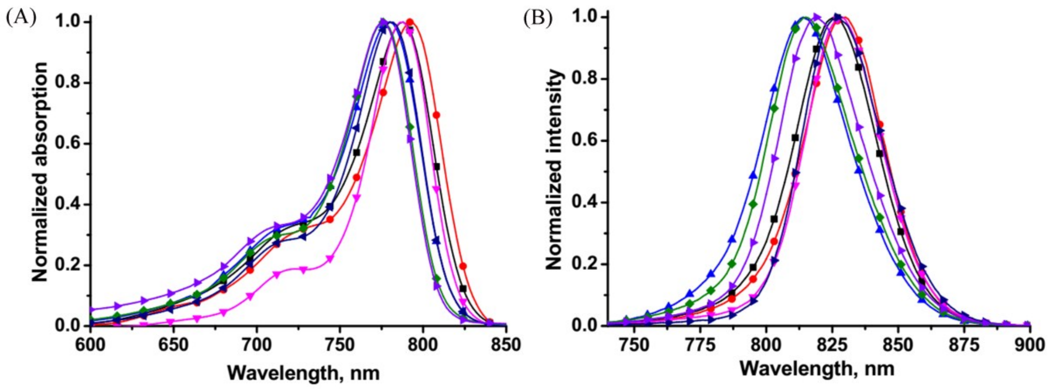

Figure S4. The absorption (A) and fluorescence (B) spectra of compound **CyCl** in distinct organic solvents, including DMF (■), DMSO (●), acetone (▲), DCM (▼), MeOH (◆), EtOH (◀), and MeCN (▶).

Table S1. Photophysical data of the dyes **CyBX** in DMF.

dye	λ_{abs}/nm[a]	ε_{max}/ $M^{-1}cm^{-1}$	λ_{em}/nm[b]	Φ_f[c]	Stokes shift/nm	Fwhm/nm[d]
**CyBO**	768	164580	805	0.328	37	41
**CyBN**	767	81050	801	0.279	34	44
**CyBS**	770	13650	799	0.026	29	48
**CyCl**	786	204420	825	0.173	37	37

[a] The maximal absorption of the dye; [b] The maximal emission of the dye; [c] Φ_f is the relative fluorescence quantum yield estimated by using ICG (Φ_f = 0.13 in DMSO) as a fluorescence standard; [d] The full width at half-maximum height.

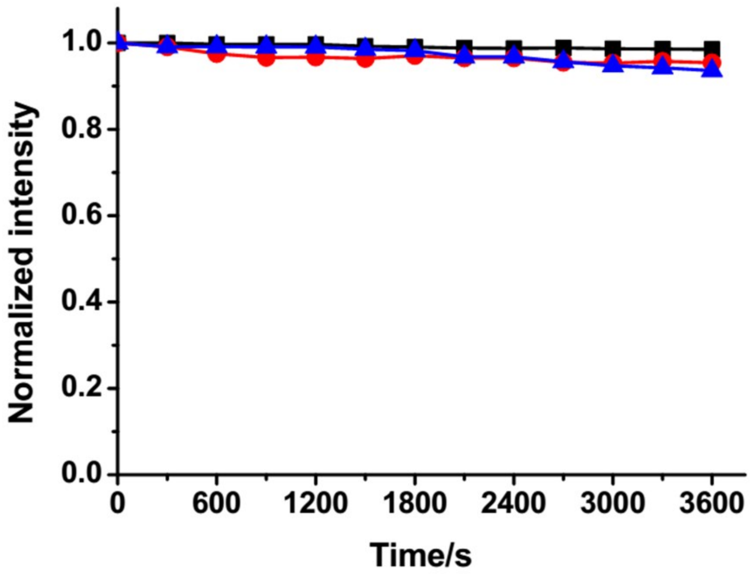

Figure S5. (A) Photostability of **CyBO** (■), **CyBN** (●), **CyBS** (▲) in DMF. The compounds were continuously irradiated by a xenon lamp (150 W) at 10 nm slit width at the corresponding maximal absorption wavelength of **CyBX**.

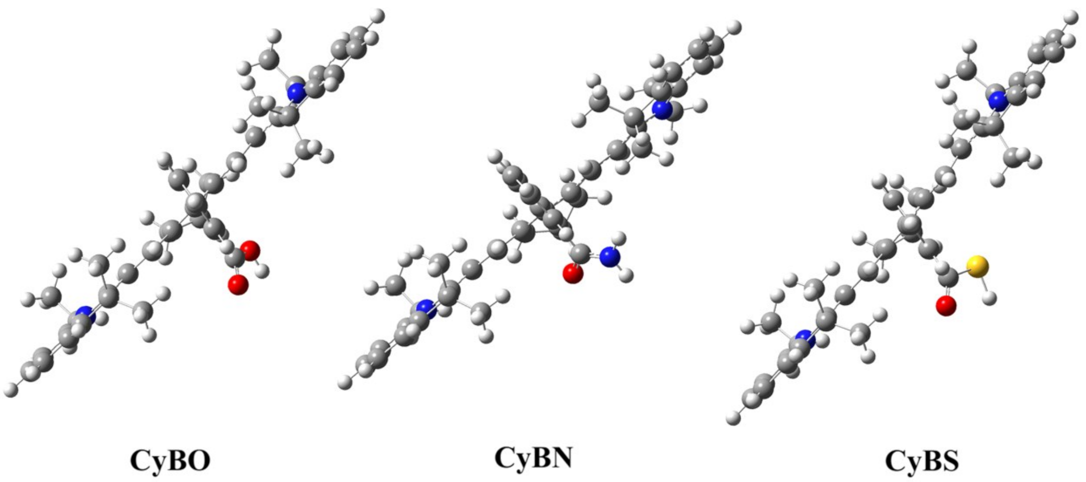

Figure S6. DFT optimized structures of **CyBX** (**X** = **O**, **N**, or **S**). In the ball-and-stick representation, hydrogen, carbon, nitrogen, oxygen, and sulphur atoms are colored in grey, gray, blue, red, and yellow, respectively.

Table S2. The dihedral angles between the benzoic acid (benzamide or benzothioic

acid) moiety and heptamethine cyanine unit.

dye	dihedral angle
**CyBO**	84.68°
**CyBN**	88.13°
**CyBS**	81.97°

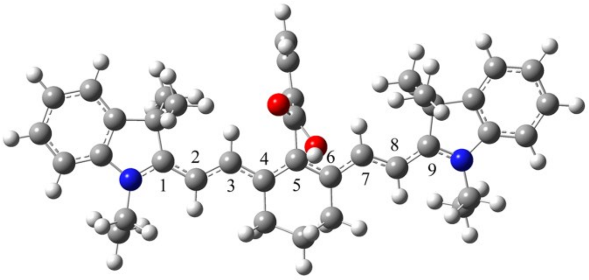

CyBO

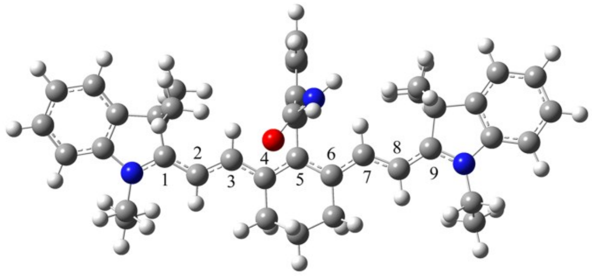

CyBN

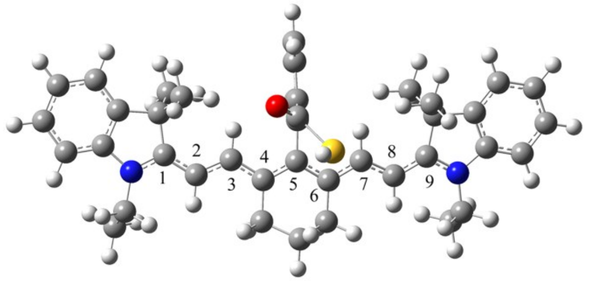

CyBS

Figure S7. DFT optimized structures of **CyBX**. In the ball-and-stick representation, hydrogen, carbon, nitrogen, oxygen, and sulphur atoms are colored in grey, gray, blue, red, and yellow, respectively.

Table S3. Representative partial carbon charges of **CyBX** (The assignment of carbon atoms is listed in Figure S6).

dye	C1	C2	C3	C4	C5	C6	C7	C8	C9
**CyBO**	0.321	− 0.341	− 0.341	− 0.063	0.049	− 0.059	− 0.128	− 0.341	0.317
**CyBN**	0.322	− 0.338	− 0.112	− 0.051	0.056	− 0.061	− 0.138	− 0.343	0.310
**CyBS**	0.325	− 0.339	− 0.131	− 0.059	0.031	− 0.060	− 0.136	− 0.340	0.318

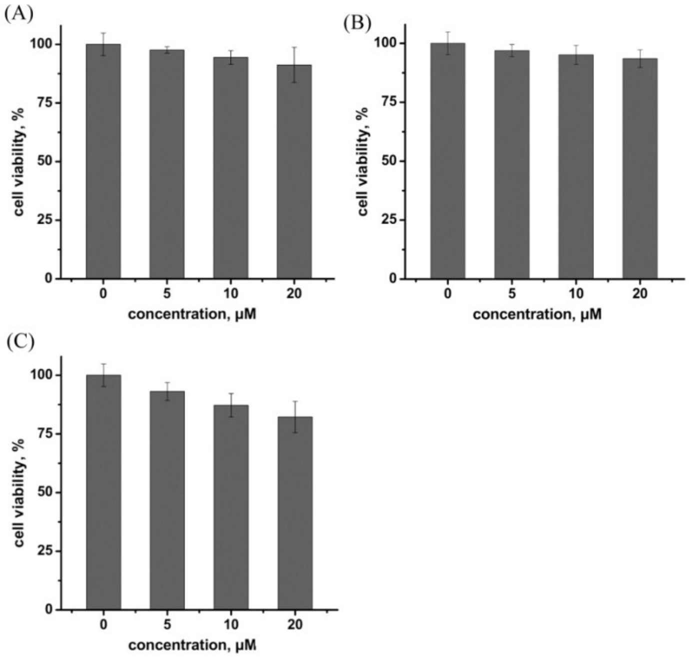

Figure S8. Cytotoxicity of (A) **CyBO**, (B) **CyBN**, and (C) **CyBS** evaluated on EC109 cells by MTT assays. The cells were incubated with the probe for 24 h.

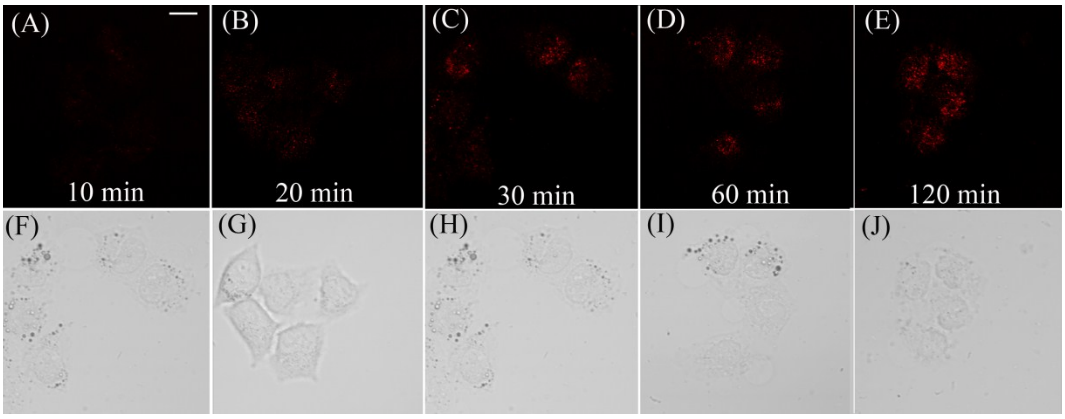

Figure S9. (A)-(E) Time dependence of the confocal fluorescence images of living cells incubated with **CyBO** (5 μM) from 10-120 min; (F)-(J) Brightfield images for (A)-(E). Scale bar = 10 μm.

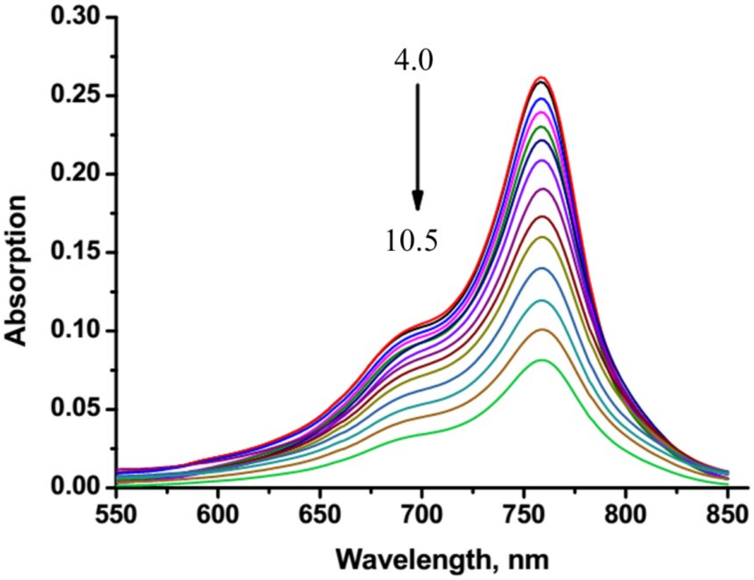

Figure S10. pH-dependence of the absorption spectra of the NIR probe **CyBN** (10 μM, DMF/PBS 5/95).

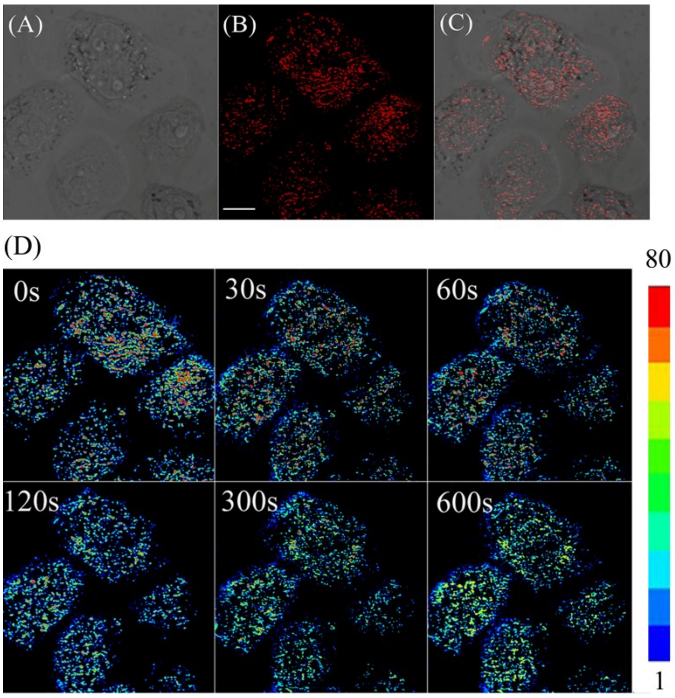

Figure S11. Images of EC109 cells treated with **CyBN**. (A) Brightfield image of EC109 cells stained with 5 μM **CyBN** in pH 7.0 PBS solution. (B) Confocal fluorescence image of (A). (C) Overlay of (A) and (B). (D) Followed by addition of 10 mM NH_4Cl, continuously imaged (B) within 10 min with *pseudo*color showing the changes of pH with time. Scale bar = 10 μm.

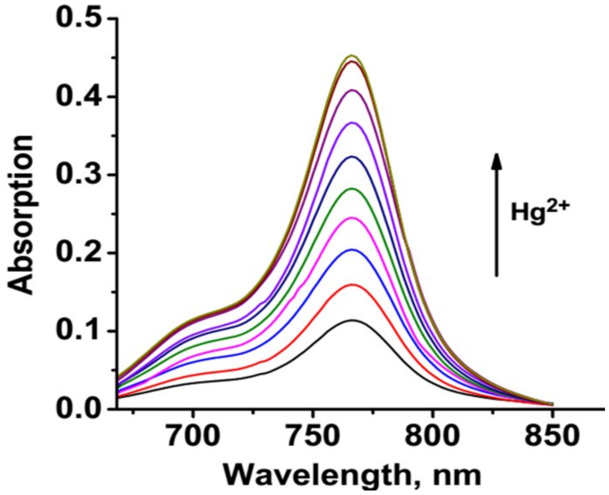

Figure S12. Absorption spectra of **CyBS** (10 μM) in the presence of various concentrations of Hg^{2+} (0 − 30 μM) in phosphate buffer (pH 7.4, 30 % MeOH).

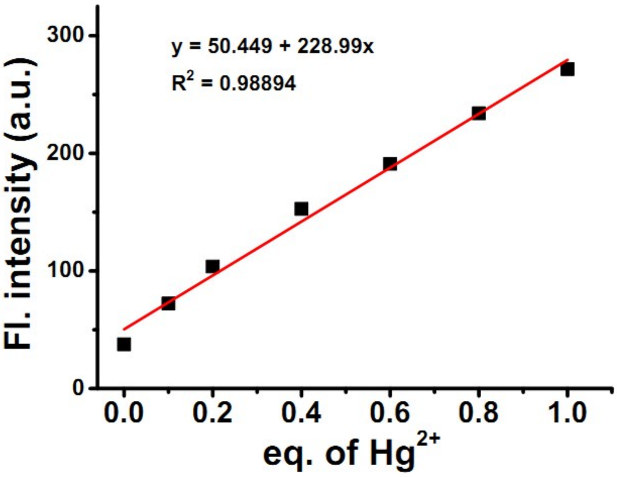

Figure S13. The linear relationship between the fluorescence intensity at 792 nm of the probe **CyBS** (10 μM) and the concentration of Hg^{2+}.

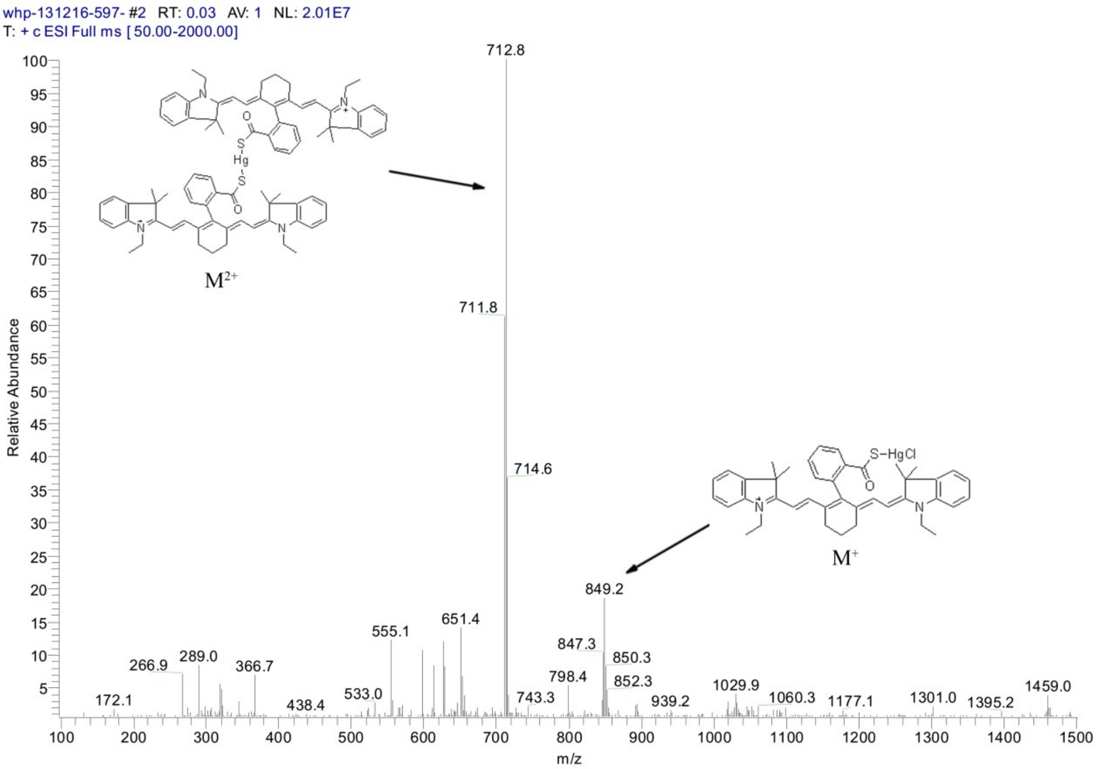

Figure S14. ESI-MS of **CyBS** in the presence of 1 equiv. $HgCl_2$ in pH 7.4 PBS-CH_3OH solution.

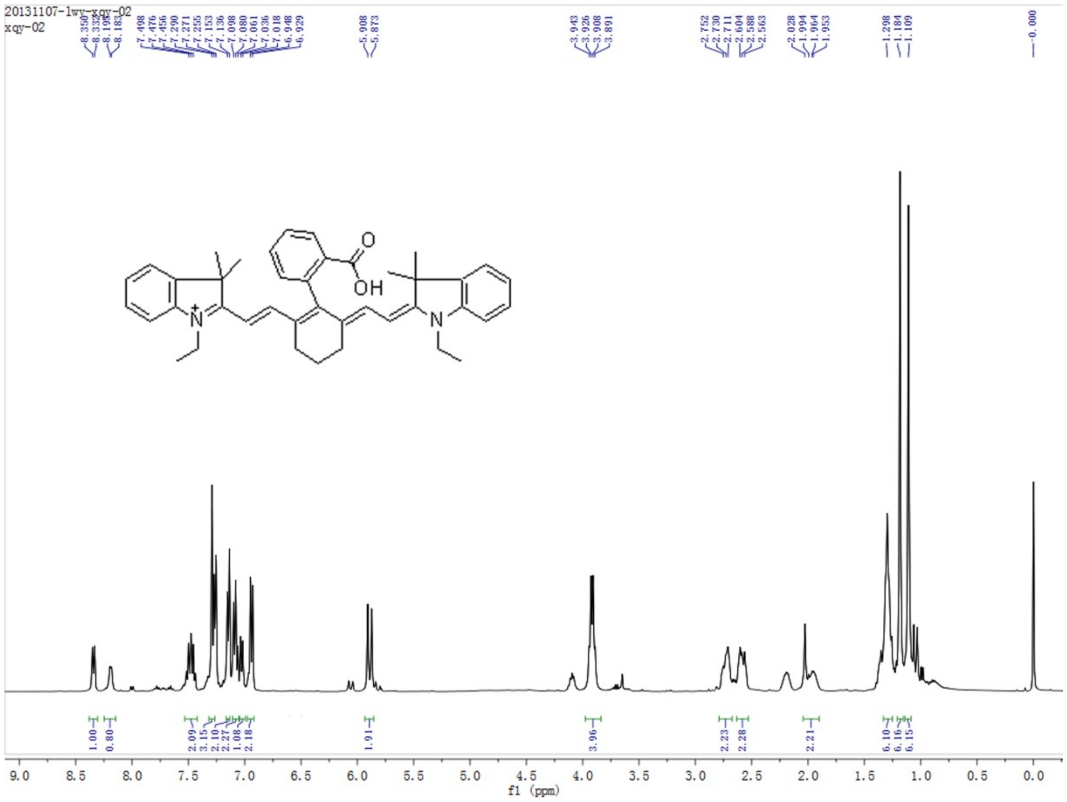

Figure S15. ^{1}H NMR spectrum of compound **CyBO** in $CDCl_3$.

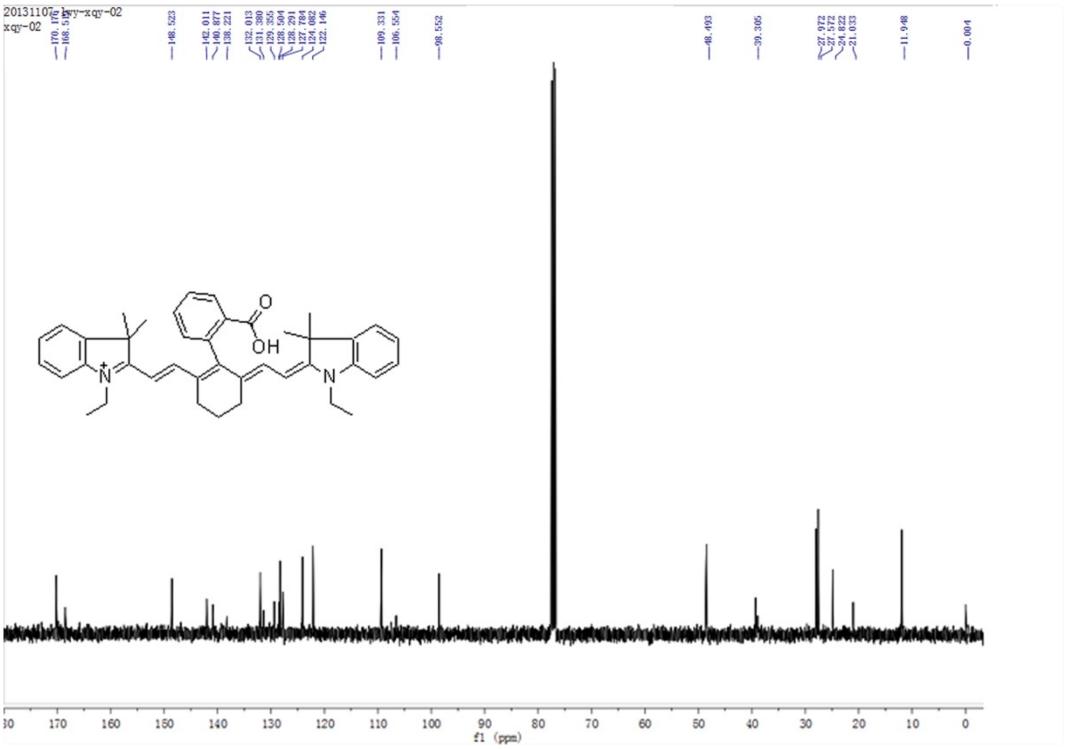

Figure S16. ^{13}C NMR spectrum of compound **CyBO** in $CDCl_3$.

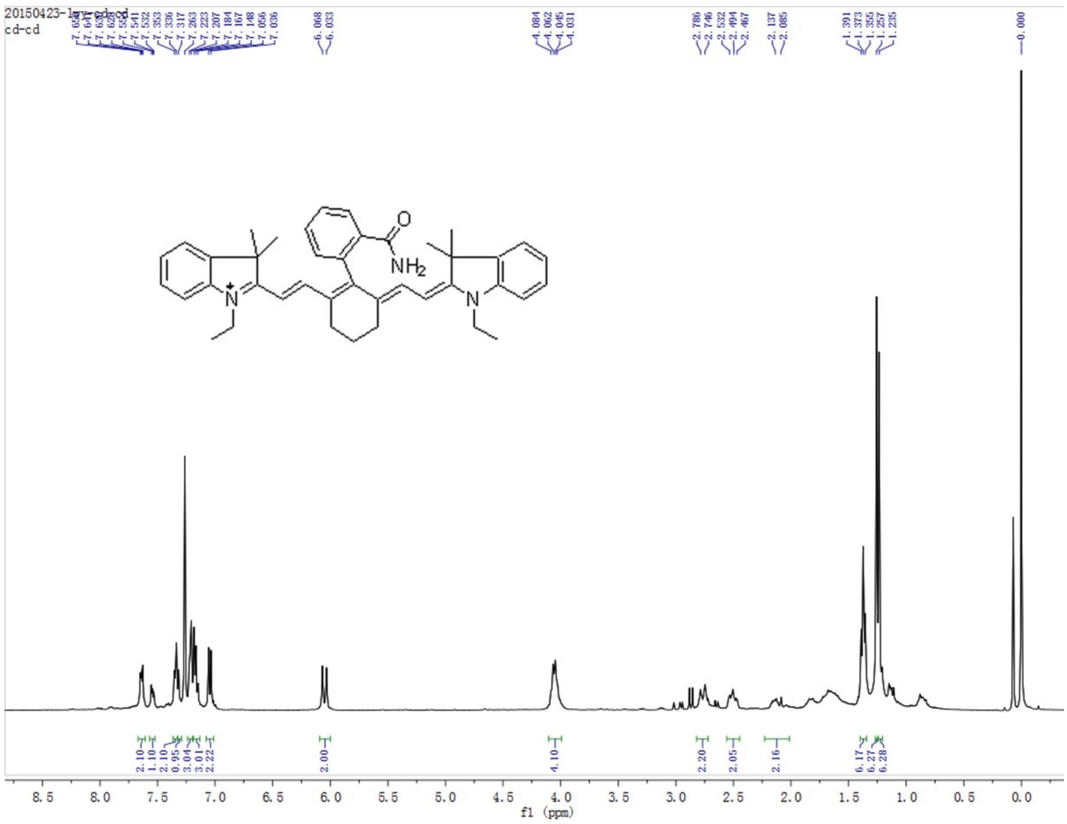

Figure S17. ^{1}H NMR spectrum of compound **CyBN** in $CDCl_3$.

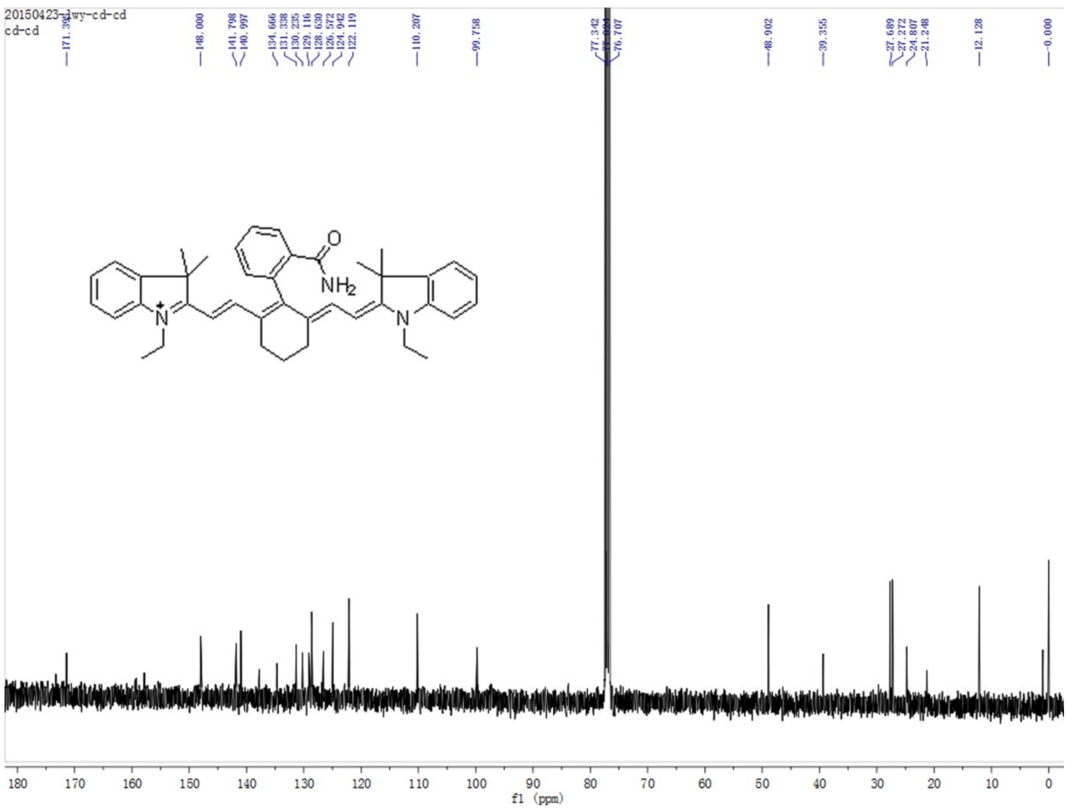

Figure S18. ^{13}C NMR spectrum of compound **CyBN** in $CDCl_3$.

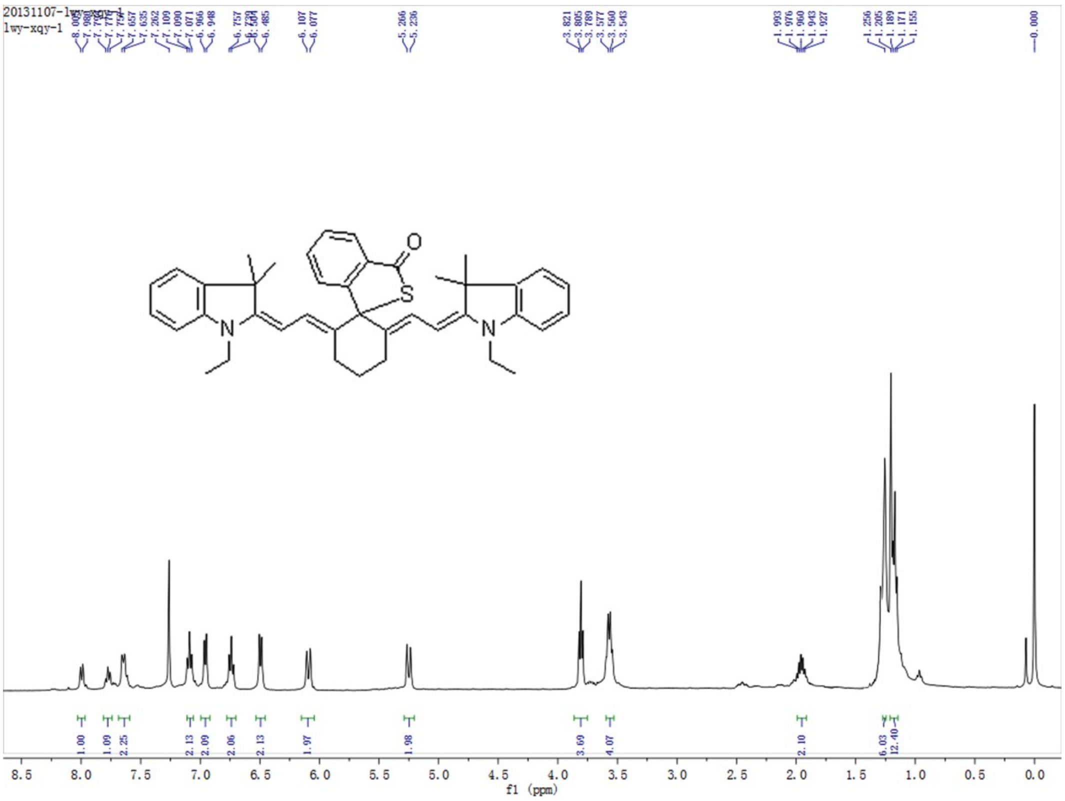

Figure S19. ^{1}H NMR spectrum of compound **CyBS** in $CDCl_3$.

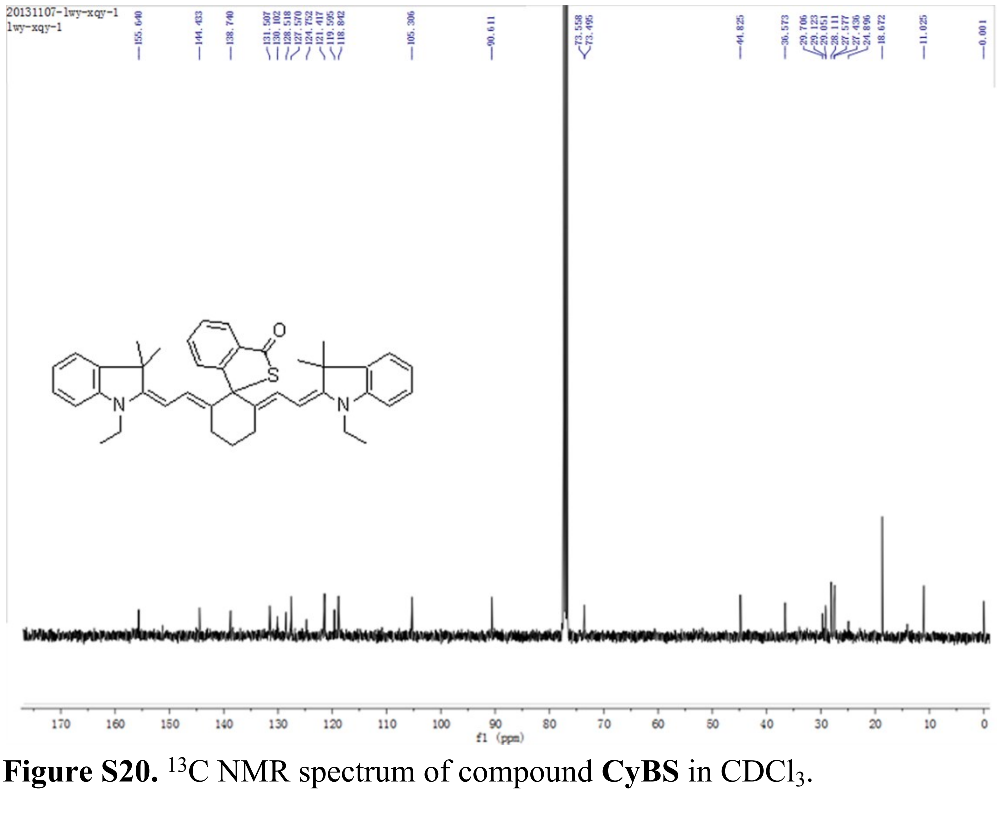

Figure S20. ^{13}C NMR spectrum of compound **CyBS** in $CDCl_3$.
